# Delayed Presentation of Concomitant Mid-ileal Mesenteric Tear and Perforation Following Blunt Abdominal Trauma: A Case Report

**DOI:** 10.7759/cureus.70412

**Published:** 2024-09-28

**Authors:** Vidhya Sree S, Balasubramanian Arumugam, Bhanumati Giridharan, Sandhya R Palit, Nikhithaa P

**Affiliations:** 1 General Surgery, Employees' State Insurance Corporation (ESIC) Medical College & Hospital, Chennai, IND; 2 Medicine, Employees' State Insurance Corporation (ESIC) Medical College & Hospital, Chennai, IND

**Keywords:** blunt abdomen trauma, delayed presentation, laparotomy, mesenteric tear, mid-ideal perforation, primary anastomosis

## Abstract

The abdomen is involved in most of the trauma cases ranging from a simple to a life-threatening one. Blunt trauma is more dangerous than penetrating trauma to the abdomen. Though blunt abdominal trauma (BAT) is common, its diagnostic difficulties lead to fatal consequences, mainly attributable to the delay in diagnosis owing to the masquerading normalcy in the index presentation of the patient. Morbidity and mortality rates of hollow viscus and mesenteric injuries are quite high per se, with diagnostic delays worsening the scenario. Successful management of the patient lies mainly in early diagnosis and treatment of the underlying bowel pathology. We hereby report a case of a 48-year-old male, who presented with a mid-ileal mesenteric tear and a separate mid-ileal perforation, 96 hours later than trauma and was managed with laparotomy and primary anastomosis.

## Introduction

Blunt abdominal trauma (BAT) is not a new entity described in medical literature, although reports of mesenteric and intestinal injuries have been uncommon. The incidence varies worldwide and is more frequent in the pediatric age group than in adults [1]. Causes of BAT include road traffic accidents, falls from heights, and assaults [1,2]. The most commonly injured organs due to BAT because of the acceleration and deceleration forces are the solid and fixed ones like the liver, spleen, and kidney. The gastrointestinal tract is often affected in penetrating rather than blunt injuries, but when it does get in the way of any blunt force, the relatively fixed points like the duodenojejunal flexure (hence, the corresponding proximal jejunum) and the ileocecal junction (hence, the terminal ileum) is easily devitalized due to the tangential forces, that directly or indirectly injure the mesentery [3]. It is difficult to identify such injuries in an acutely injured patient, in the absence of other examination findings like the seatbelt sign which might be missed unless looked for, along with other nonspecific findings like hypotension [4,5]. The fact that, at the time of presentation to the emergency room with other distracting injuries, the patient has a normal bowel function despite a mesenteric injury (which is waiting to implode into an intestinal rupture) makes it challenging to diagnose this condition while posing a greater threat to the patient’s life [6]. We hereby report the case of a middle-aged male, who had a delayed presentation of intestinal perforation at an unconventional site, successfully treated with surgical intervention.

## Case presentation

A 48-year-old male presented to our Emergency Department (Employees' State Insurance Corporation {ESIC} Medical College and Hospital, Chennai), with complaints of abdomen pain and distension (since the past 36 hours, increasing severity with time). He had met with a road traffic accident four days back. He was hit (by a direct blow to his right hip and his right-sided abdomen) by a three-wheeler (motor tricycle traveling at a speed of 40 km/h) while he tried to cross the road as a pedestrian. As a result, he had a brief loss of consciousness for a few seconds. He was promptly taken to a nearby primary health care center for initial medical assistance. He initially had complaints of dull aching pain over his abdomen and back, and headache. During the examination, there were no neurological deficits observed (Glasgow Coma Scale {GCS} score of 15/15). He displayed no abrasions, contusions, or lacerations on his face, and there were no indications of external injuries elsewhere on his body. His entire abdomen was soft to the touch, with no signs of guarding or rigidity. Bowel sounds were normal, and there was only slight diffuse tenderness upon deep palpation. Furthermore, no other indications of thoracic or abdominal injuries were noted. His chest X-ray and abdominal X-ray (erect and supine films) showed no air under the right hemi-diaphragm or any fractures. He was observed for about four hours, given IV fluids and analgesics, and then discharged with caution, that he might need further imaging like a CT scan if he experienced any pain in the abdomen or other symptoms of peritonism. Following administration of analgesics, the patient experienced a reduction in headache and abdominal pain. During the initial 72 hours after being discharged, the patient maintained normal bowel and bladder functions at home. However, beyond this period, there was a gradual escalation in the intensity of abdominal pain, accompanied by a progressive development of abdominal distension. Although the patient initially tolerated the first diet after the onset of distension, subsequent episodes were marked by nausea and an inability to tolerate oral feeds. He then chose to take a CT abdomen on the advice of his initial health provider while on the way to our institute. The patient was a chronic alcoholic but claimed that he had not consumed it at the time of the accident. He did not have any comorbid conditions. 

In our institute, initial examination revealed the patient to have a normal airway, tachypnea (respiratory rate - 30 per minute), tachycardia (pulse rate - 120 per minute), hypotension (systolic 100 mmHg, diastolic - 60 mmHg); conscious, oriented, with some dehydration and had a normal axillary temperature. GCS was 15 out of 15. Per abdomen examination, findings were: diffuse tenderness, uniform distension, central guarding, and absent bowel sounds. The patient was started on IV fluid resuscitation via a large bore cannula, nasal oxygen support was given, a nasogastric tube and a urinary catheter were inserted; blood and urine samples were collected and sent for investigations (Table 1).

**Table 1:** Abnormal parameters summarized

S. No.	Parameter	Patient value	Reference range
1.	Hemoglobin	10 g/dl	12-16 g/dl
2.	Total cell count	19700 /cu.mm	4000-11000 /cu.mm
3.	Serum urea	50 mg/dl	17-43 mg/dl
4.	Serum creatinine	2.3 mg/cl	0.7-1.2 mg/dl

CT abdomen plain showed gross pneumoperitoneum without any solid organ injury; CT brain showed an old infarct (subdural infarct in the posterior limb of internal capsule). We are unable to provide any of those images here mainly because both the imaging were done elsewhere and we don't have records of the same.

**Figure 1:**
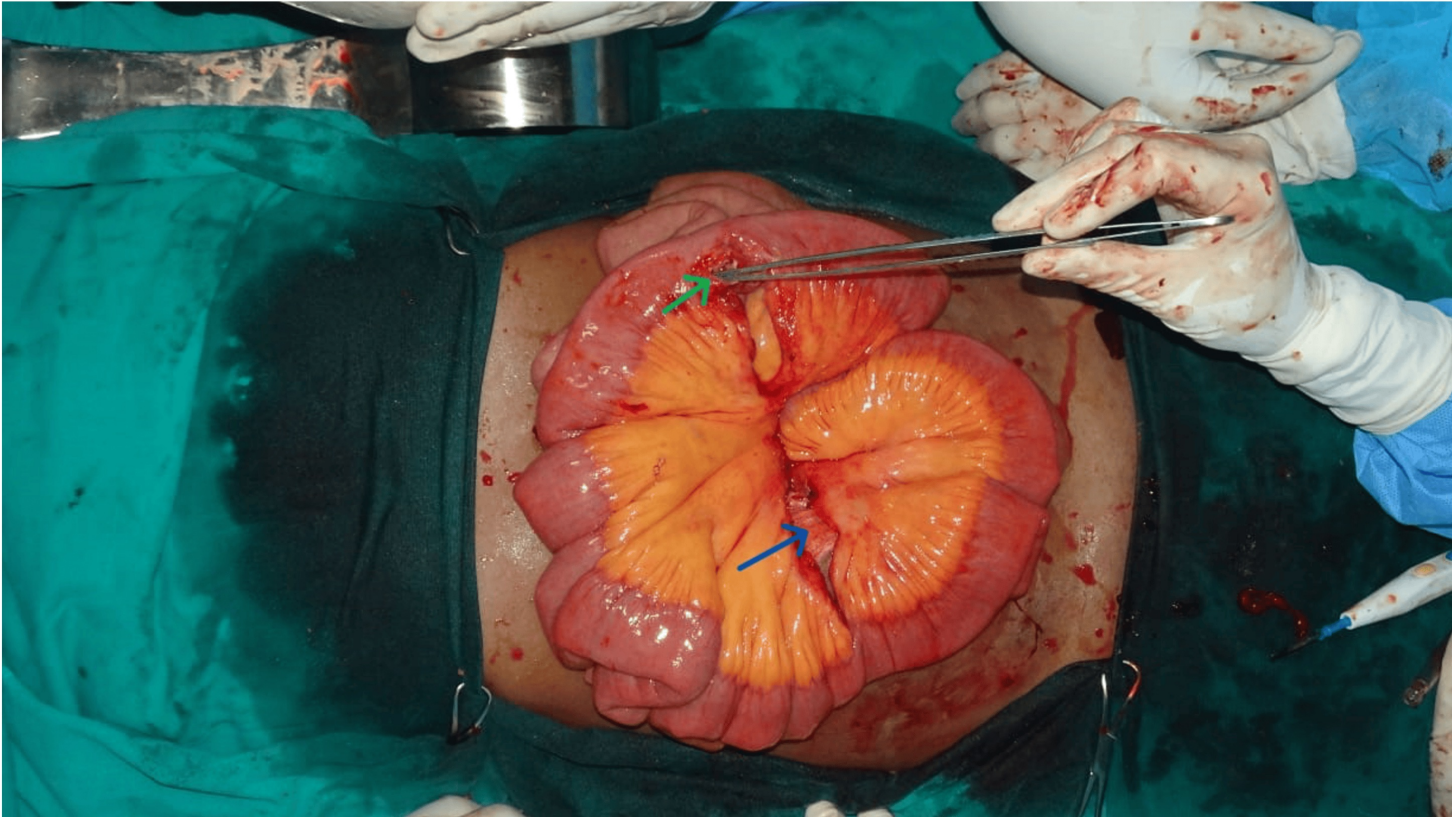
Ileal perforation, 2.8 meters proximal to the ileocecal junction (green arrow); mesenteric tear corresponding to the mid-ileal level 1.5 meters proximal to the ileocecal junction (blue arrow).

With informed written consent, the patient was taken up for emergency exploratory laparotomy under general anesthesia (Table 2). Thorough peritoneal lavage was given. Segmental resection of the perforated ileal segment and the area of the ileum corresponding to the mesenteric tear was done (Figure 1), followed by a primary handsewn ileoileal anastomosis at both sites.

**Table 2:** Intraoperative findings summarized

S. No.	Intraoperative findings
1.	Hemo- (1.5 liters of dark red collected blood) and pneumoperitoneum
2.	An ileal perforation which was 2.8 meters proximal to the ileocecal junction
3.	A mesenteric tear corresponding to the mid-ileal level 1.5 meters proximal to the ileocecal junction with the corresponding bowel being pale and paralytic
4.	All solid organs were normal
5.	No retroperitoneal injury

Postoperatively, broad-spectrum IV antibiotics were given (seven-day course), a packed cell transfusion was done and ICU care was given. On the second postoperative day, the patient developed basal atelectasis, which was managed adequately with nebulization and chest physiotherapy. By the third day following the operation, the patient's bowel functions had returned to normalcy and his blood parameters returned to normal limits. At this point, he was cleared for oral intake starting with liquids, and the urinary catheter was removed. On the fifth postoperative day, the drain was removed. The patient was discharged on the seventh day when he was tolerating solids well, and scheduled for a follow-up appointment on the 10th day for the removal of skin sutures.

## Discussion

The prevalence of intra-abdominal injuries amongst patients presenting to the emergency department is approximately 13% [5]. Traumatic hollow viscus and mesenteric injuries (HVMI) are reported in approximately 1.2% of blunt trauma and 17% of penetrating trauma [7]. Generally, the incidence of trauma is higher in males, but there is a universal variation in the distribution of cause according to the age groups affected. The odds of death increase by 1% for every three minutes spent in the emergency room [8]. HVMI is associated with a higher mortality and complication rate when compared to patients with similar Injury Severity Score (ISS) but without HVMI [9-11]; this is due to the delay in diagnosis in-turn, due to the delay in presentation of the warning signs and symptoms [4].

The initial objective is to ascertain the mechanism of injury in patients with trauma, as this will provide valuable insights into the potential types of injuries that can be expected. Patients might have any of the physical examination findings ranging from a simple bruise to the most significant “seatbelt sign;” a positive sign is linked to a 12% chance of bowel injury [12]. Serum procalcitonin and compensatory reserve measurement are found to be more sensitive than other measurements of vital signs, such as white cell count, C-reactive protein, and qSOFA (quick sequential organ failure assessment) [13,14]. The presence of other critical injuries like an intracranial hemorrhage, a spinal cord syndrome, or a pelvic fracture should not deter a surgeon from ruling out an intestinal injury [4,9]. While the initial school of thought suggested that solid organs were the primary targets of BAT, a more widely embraced viewpoint now acknowledges the mesentery as the instigator of bowel perforations. Mesenteric lacerations can be attributed to the interplay of shear and tangential forces, traction, and counter-traction, resulting in stretching and subsequent rupture of the mesentery [6]. A mesenteric hematoma or a sealed subclinical bowel perforation may trick the surgeon into believing that there is no indication to withhold enteral feeding [4,6]. 

Management of a conscious BAT patient starts with a primary survey, e-FAST (extended focused assessment with sonography for trauma), physical examination, secondary survey, and blood chemistry followed by a contrast-enhanced abdominal CT scan, i.e., a trauma scan [4]. When performed by an experienced sonologist, the identification of intraperitoneal air in ultrasound, which serves as a conclusive indicator of bowel injury, offers a distinct advantage to the patient [15]. The trauma scan also has been proven notorious for missing certain injuries initially, 20% of which are injuries to the intestines [16]. According to Bonomi et al., a definitive candidate for laparotomy can be identified using a contrast-enhanced CT scan if the patient exhibits four or more out of the following six criteria (with a sensitivity of 98%): (a) free fluid without solid organ injury, (b) free intraperitoneal air, (c) gastrointestinal wall alteration (any focal anomaly of the bowel wall, thickening or thinning, abnormal or lack of enhancement with contrast, (d) mesenteric alteration (mesenteric hematomas and fat stranding), (e) intra-mesenteric fluid (accumulating between mesenteric layers and assuming a typical triangle aspect), (f) mesenteric blushing (active leak of intravenous contrast) [9]. 

Hence, it is better to admit the seemingly normal patient, with non-specific imaging findings after having suffered a high-risk mechanism of injury, and observe for a worsening clinical condition [17]. Hesitating to repeat an investigation can be deadly; the frequency of repetition should generally be eight hours for blood investigations and six hours for a trauma scan [4,9]. A comatose polytrauma patient is better justified in being taken up for exploratory surgery, the choice between laparoscopy and laparotomy being dependent on the hemodynamic stability of the patient; the former is a contraindication in case of an unstable and deteriorating patient [3-5,18]. Opting for non-operative management in a hemodynamically stable patient [9,11] is a tough call to make, especially after the imaging reveals a mesenteric hematoma; in such a case, a rigorous follow-up of the patient should be carried out after discharge [4,6]. 

Over the past two decades, diagnostic laparoscopy has increasingly emerged as a valuable instrument for both diagnosing and treating intestinal injuries; in a hemodynamically stable patient, it allows for a timely diagnosis and prompt treatment avoiding diagnostic delay and unnecessary laparotomy [3,4]. The benefits of laparoscopy are reduced postoperative surgical site complications, pneumonia and length of hospital stays [19,20]. The gold standard treatment of choice is and will be laparotomy; the choice should always be made to primarily anastomose (superiority of techniques not sufficiently proven yet) the small bowel, thanks to its greater resilience and abundant healing capacity [4,21], more so in patients who present like ours presenting with no signs of peritonitis, despite a delay. The leak rates have been found to be <3% in various studies; the leading factors include time from initial surgery, ongoing transfusion requirements, ongoing inotropic support, tissue edema, intra-abdominal sepsis, and time to abdominal fascial closure [4].

## Conclusions

A high suspicion of bowel injury can save a BAT patient from terminal consequences. No single sign is completely reliable, nor any imaging gold standard can rule out a HVMI. CT should be used as a screening tool to look for pointers. Decision to discharge a patient should be made after weighing the risks, with all the warning signs well explained beforehand and the conservatively managed patient should be reviewed in the OPD for weeks after trauma. Though there have been reports of proximal jejunal and terminal ileal tears due to BAT, our patient had a mid-ileal perforation which makes it a novel finding to report.
